# Cross-cohort meta-analysis reveals conserved gut microbiome signatures of insomnia

**DOI:** 10.1016/j.crmicr.2026.100577

**Published:** 2026-03-06

**Authors:** Sun Park, Jiseon Yang, Se-Hui Lee, Juyoung Jeong, Ju-Yeon Jang, Jennifer Barrila, Jin-Young Yang

**Affiliations:** a Department of Integrated Biological Sciences, Pusan National University, Busan 46241, Republic of Korea; b Biodesign Center for Fundamental and Applied Microbiomics, Arizona State University, Tempe, AZ, USA; c School of Life Sciences, Arizona State University, Tempe, AZ, USA; d Institute for Future Earth, Pusan National University, Busan 46241, Republic of Korea; e Department of Biological Sciences, Pusan National University, Busan 46241, Republic of Korea

**Keywords:** Gut microbiome, Insomnia, Microbial functional pathway, Gut-brain axis, Immune-microbiome interaction, Meta-analysis

## Abstract

Insomnia is a prevalent sleep disorder associated with broad metabolic, immune, and neuropsychiatric consequences, yet its association with the gut microbiome remains difficult to define across individuals. Here, we performed a cross-cohort meta-analysis of five publicly available human fecal 16S rRNA case-control datasets to identify conserved microbiome features associated with insomnia. All raw sequencing data were reprocessed using a unified analytical workflow, generating a harmonized cohort of 468 individuals while controlling for cohort-specific effects. Across cohorts, insomnia was associated with directionally consistent differences in gut microbiome structure, including a statistically significant increase in Shannon diversity (*p* = 0.003) and a significant group effect after covariate adjustment. Integrating four complementary statistical approaches, we identified a core set of eight gut taxa that were consistently associated with insomnia across analyses. Functional prediction and integrative filtering indicated that insomnia-related microbial changes extended beyond taxonomy, with coordinated shifts in metabolic potential encompassing pathways previously linked to host immune and neurobiological processes, thereby suggesting relevance to biological systems implicated in sleep regulation. Notably, these functional alterations were concentrated within a limited subset of taxa rather than broadly distributed across the community. Among them, the Burkholderia–Caballeronia–Paraburkholderia complex emerged as a dominant functional contributor. Independent correlation analyses validated key taxon-function relationships and reinforced a model of focused, pathway-specific microbiome remodeling. Together, these findings demonstrate that insomnia is associated with a reproducible gut microbiome signature characterized by targeted functional remodeling driven by specific microbial taxa. This study provides a cross-cohort framework for investigating microbiome-mediated metabolic and immune contexts relevant to sleep regulation.

## Highlights

• Cross-cohort meta-analysis reveals reproducible gut microbiome signatures of insomnia.
• Eight conserved gut taxa are consistently associated with insomnia across studies.
• A small number of gut microbes drive most insomnia-associated functional shifts.
• The Burkholderia*-*Caballeronia-Paraburkholderia (BCP) complex drives key functions.
• Microbial metabolic changes may indirectly shape host immune and neural states.

## Graphical abstract

**Figure:**
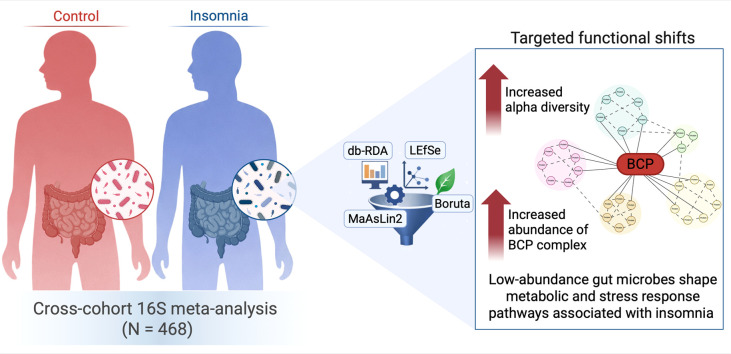

## Introduction

Insomnia is one of the most prevalent sleep disorders worldwide and is associated with impaired daytime functioning, cognitive deficits, and emotional dysregulation, resulting in substantial adverse impacts on quality of life and socioeconomic burden (Benjafield et al., 2025). Current therapeutic approaches include first-line treatment with Cognitive Behavioral Therapy for Insomnia (CBT-I), which primarily targets behavioral changes (Qaseem et al., 2016). Pharmacological options, such as benzodiazepines and non-benzodiazepine receptor agonists (Z-drugs), are typically intended for short-term use (Burry et al., 2022). Despite their clinical utility, these approaches do not directly address the underlying biological mechanisms of chronic insomnia, highlighting persistent challenges in long-term management and the need for novel therapeutic strategies.

Emerging evidence suggests that insomnia reflects a state of sustained physiological hyperarousal involving coordinated dysregulation of neuroendocrine, metabolic, and immune pathways (Cryan et al., 2019; Irwin et al., 2015; Riemann et al., 2011). Within this framework, the gut microbiome has gained attention as a potential modulator of host systems relevant to sleep regulation (Cryan et al., 2019). The gut microbiome plays a central role in host metabolic and immune regulation and communicates with the central nervous system through the gut–brain axis via microbial metabolites, immune mediators, and neuroendocrine pathways (Cryan et al., 2020). Microbial-derived metabolites, including short-chain fatty acids (SCFA), influence epithelial integrity, immune tone, and hypothalamic-pituitary-adrenal axis activity, which are relevant to sustained arousal and stress responsiveness (Dalile et al., 2019; Furusawa et al., 2013). Given that insomnia is defined by persistent hyperarousal rather than acute inflammation, microbiome-associated effects are expected to be modest and mediated through systemic regulatory processes rather than overt immune activation (Riemann et al., 2011). This biological framework has motivated multiple human cohort studies examining associations between insomnia and the gut microbiome.

Multi-human cohort studies have examined associations between insomnia and the gut microbiome, predominantly using 16S rRNA gene sequencing. Case-control studies have reported differences in microbial community structure between individuals with insomnia and healthy controls, including alterations in alpha and beta diversity as well as taxon-level shifts (Li et al., 2020; Liu et al., 2019). Several cohorts have observed reduced abundance of putative short-chain fatty acid-producing genera, such as *Faecalibacterium* and *Lachnospira*, alongside increased abundance of taxa including *Blautia* and members of the *Bacteroidales* (Haimov et al., 2022; Li et al., 2020; Liu et al., 2019). Other studies have identified insomnia-associated differences in *Ruminococcus, Prevotella*, and *Bacteroides*-related taxa, although the specific taxa and directions of change vary across cohorts (Haimov et al., 2022; Liu et al., 2019, Liu et al., 2022).

Beyond taxonomic composition, functional inference analyses using tools such as PICRUSt2 have suggested alterations in microbial pathways related to amino acid metabolism and stress-associated signaling, with predicted functional profiles showing modest associations with insomnia severity in some cohorts (Douglas et al., 2020; Liu et al., 2019). More recently, Mendelian randomization analyses leveraging large genome-wide association datasets have provided preliminary genetic evidence for bidirectional associations between specific bacterial taxa and insomnia risk, suggesting potential causal links while also highlighting substantial heterogeneity across analyses (Yang et al., 2023). Despite the growing number of original cohort studies, findings across insomnia-microbiome investigations remain inconsistent. Reported results differ in whether overall diversity is altered, which taxa are enriched or depleted, and whether associations persist after covariate adjustment. These discrepancies likely reflect heterogeneity in cohort composition, including limited sample sizes, population-specific recruitment, and variable diagnostic criteria for insomnia. Importantly, many studies have incompletely controlled for major confounders known to influence gut microbiome composition, such as diet, geographic location, psychiatric comorbidities including depression and anxiety, and medication use. Inconsistent adjustment for sleep medications, antidepressants, and anxiolytics represents a critical limitation, given their direct effects on both sleep physiology and microbial communities.

Methodological heterogeneity further complicates reproducibility and cross-cohort comparability across prior insomnia–microbiome studies. Different analytical approaches capture distinct aspects of microbiome–phenotype associations, as no single method is sufficient to robustly capture these relationships in heterogeneous human cohorts. For example, distance-based redundancy analysis (db-RDA) enables multivariate assessment of community-level variation while accounting for explanatory variables, but does not identify the specific taxa driving observed differences (Legendre and Anderson, 1999). Differential abundance approaches, such as LEfSe, facilitate the identification of discriminative taxa with effect-size estimation, yet are sensitive to compositionality and cohort-specific effects and may yield non-reproducible signatures when applied in isolation (Gloor et al., 2017; Nearing et al., 2022; Segata et al., 2011). Multivariable association models such as MaAsLin2 allow adjustment for key clinical and demographic covariates, including age, medication use, and psychiatric comorbidity; but are sensitive to sample size and model specification and may miss weak but coordinated signals(Mallick et al., 2021). Feature selection methods such as Boruta provide a complementary, model-agnostic approach to identify robust predictive features in high-dimensional microbiome data, but do not directly infer statistical associations or directionality (Kursa and Rudnicki, 2010). Finally, functional inference tools based on 16S rRNA gene profiles, including PICRUSt2, enable hypothesis generation regarding potential metabolic and regulatory pathways but rely on reference-based predictions that may not reflect in vivo activity (Douglas et al., 2020). Integrating these complementary analytical approaches within a unified framework facilitates the differentiation of reproducible, cohort-independent microbiome features from study-specific artifacts and enables a more robust characterization of conserved microbiome signatures associated with insomnia.

Accordingly, it remains unclear whether insomnia is associated with conserved gut microbiome features that persist across independent cohorts, or whether previously reported associations primarily reflect study-specific variability arising from technical, demographic, and analytical heterogeneity. To address these gaps, we performed a cross-cohort meta-analysis of publicly available human fecal 16S rRNA datasets. By integrating multiple independent insomnia cohorts under a unified analytical framework, we systematically reduce technical and cohort-specific heterogeneity. This integrative strategy enables the identification of gut microbiome features that are consistently associated with insomnia across populations. We further apply this framework to examine the potential functional implications of these conserved microbiome alterations. Collectively, this study identifies a reproducible gut microbiome signature of insomnia and provides a foundation for understanding its functional relevance.

## Materials and methods

### Study design and data sources

We performed a systematic search for human fecal 16S rRNA gene sequencing studies with a case-control design comparing individuals with insomnia and non-insomnia controls. PubMed was queried programmatically using Biopython, and retrieved records were screened and managed in Python using pandas (Cock et al., 2009; McKinney, 2011). Eligible studies included adult cohorts that reported both insomnia and control groups, provided raw sequencing reads with sample-level metadata, and contained sufficient covariate information to adjust for age, study location, and cohort. We excluded shotgun metagenomic-only studies, intervention trials without baseline case-control data, studies with non-comparable phenotypic definitions, and samples that failed downstream quality control. The original cohorts excluded individuals actively using sleep medications or major psychotropic drugs at the time of sampling.

### Read processing and taxonomic profiling

To ensure consistency across cohorts, all raw sequencing reads were reprocessed using a unified pipeline. Region-specific technical variability was minimized by removing study-specific primers and applying amplicon-matched reference classifiers before downstream analyses. Sequence processing was conducted in QIIME2, with denoising, quality trimming, error modeling, and chimera removal performed using the q2-dada2 plugin (Bolyen et al., 2019). Non-bacterial sequences were filtered prior to downstream analysis. Amplicon sequence variants (ASVs) were taxonomically classified using a naïve Bayes classifier trained on the SILVA 138.1 reference database, tailored to the amplicon region used in each study (Quast et al., 2013). Samples with fewer than 10,000 retained reads were excluded from further analyses.

### Community summaries and diversity analysis

For compositional analysis, feature tables were normalized within samples using total sum scaling. Statistical models were applied to centered log-ratio (CLR)-transformed data, whereas visualizations were generated from relative abundance profiles. Alpha diversity was calculated using the Shannon index on rarefied feature tables. Beta diversity was assessed using Bray-Curtis dissimilarities and visualized by principal coordinates analysis (PCoA). Group differences were tested using permutational multivariate analysis of variance (PERMANOVA), with insomnia status as the primary factor and age, study location, and cohort included as covariates. Permutations were constrained within cohorts. Phylum-level community composition was summarized using stacked bar plots, and the ratio of Bacillota to Bacteroidota was compared between groups using covariate-adjusted linear models. To improve interpretability, taxa with relative abundance below 0.1 % or present in fewer than 10 % of samples were aggregated into an “Other” category.

### Differential analysis

Associations between microbial taxa and insomnia were evaluated using four complementary analytical approaches implemented in R and Python. First, multivariable linear models were fitted using Microbiome Multivariable Associations with Linear Models (MaAsLin2) on transformed relative abundance data, adjusting for age, study location, and cohort (Mallick et al., 2021). Second, distance-based redundancy analysis (db-RDA) was performed on Bray-Curtis distance matrices to assess the contribution of insomnia status while accounting for covariates (Oksanen et al., 2013). Third, Boruta feature selection was applied to identify stable predictors of group status (Kursa and Rudnicki, 2010). Fourth, Linear Discriminant Analysis Effect Size (LEfSe) was used to detect taxa differentially enriched between groups (Segata et al., 2011). A consensus set of taxa was defined as those that were significant in pooled MaAsLin2 models after false discovery rate correction (*q* ≤ 0.05) and supported by all four methods. The direction and magnitude of associations were determined from the covariate-adjusted linear models. Figures were generated using GraphPad Prism (GraphPad Software, San Diego, California).

### Functional analysis and taxon-function links

Predicted functional profiles were inferred from 16S rRNA gene data using PICRUSt2, yielding Kyoto Encyclopedia of Genes and Genomes (KEGG) ortholog (KO) abundance tables aligned with the taxonomic profiles. Before testing for associations, we replaced zeros with multiplicative replacements and applied CLR transformations. We calculated taxon-function correlations using Spearman’s rank correlation between each genus and each KO across samples, adjusted p-values with the Benjamini-Hochberg method, and considered q ≤ 0.05 as significant; summaries focused on links with |ρ| ≥ 0.3. We modeled KO abundances with MaAsLin2, using Group as the main term, along with age, study location, and cohort as covariates. We compared the sign of the Group coefficient to the univariate log2 fold change (log_2_FC; Insomnia vs Control) from the same data. KOs included in displays met the criteria of |β| ≥ 0.5 and |log_2_FC| ≥ 1 with consistent direction.

### KO contribution and network view

For prioritized KOs and consensus genera, PICRUSt2 contribution scores were used to estimate the contribution of individual taxa to each KO. Bipartite taxon-KO networks were constructed and visualized in Cytoscape, with node colors indicating the group in which adjusted abundances were higher among connected features.

## Results

### Cross-cohort meta-analysis identifies gut microbiome alterations associated with insomnia

**Fig. 1:**
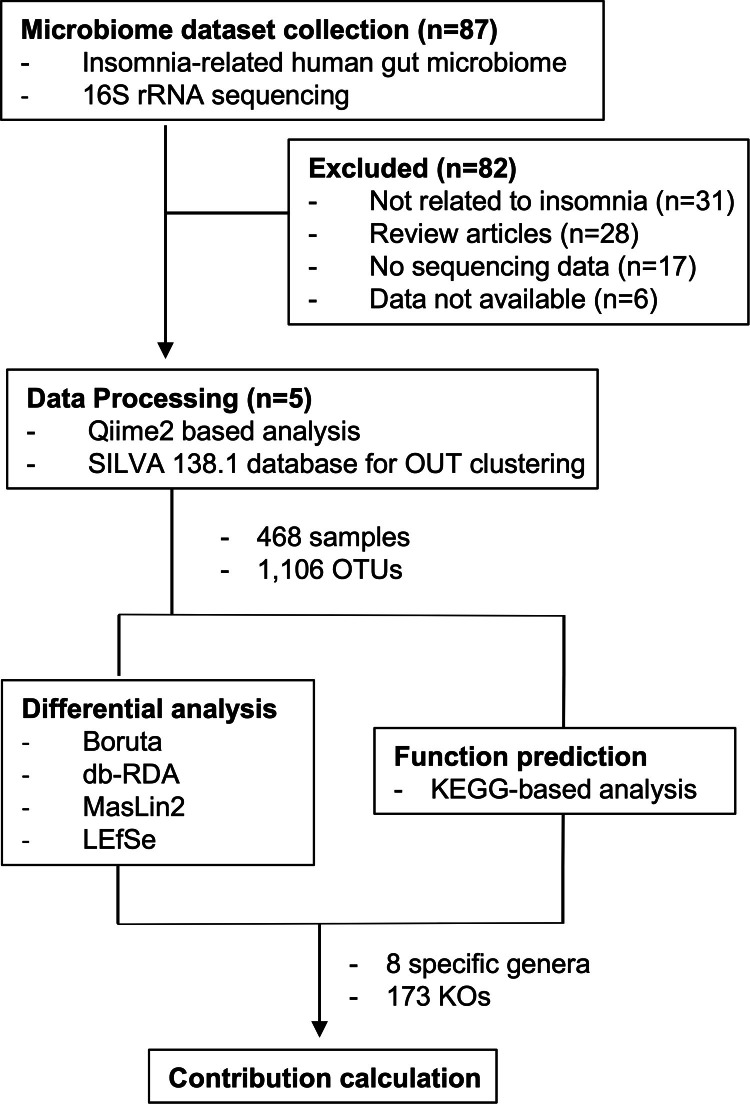
**Overview of the multi-omic meta-analysis pipeline for the insomnia-associated gut microbiome.** Schematic representation of the integrated workflow combining multi-cohort microbiome datasets, followed by independent multi-omic cohort validation. The pipeline encompasses standardized preprocessing, statistical meta-analysis, functional inference, and cross-cohort integration to identify reproducible microbial and functional signatures associated with insomnia.

**Fig. 2:**
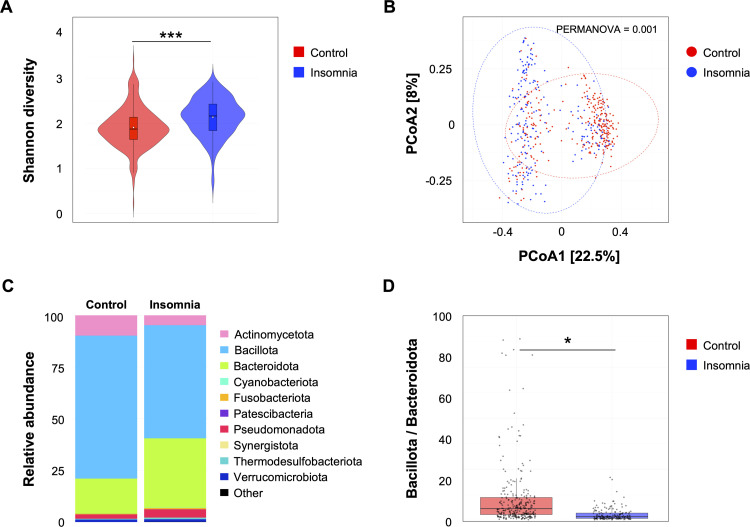
**Insomnia exhibits higher alpha diversity and significant community separation.** (**A**) Shannon diversity calculated on rarefied ASV tables is shown as violin plots with embedded boxplots displaying the median and interquartile range. Group comparison was performed using an unpaired two-sided *t*-test (p = 0.003). (**B**) Principal coordinates analysis (PCoA) based on Bray-Curtis dissimilarity, visualized with group centroids and 95 % confidence ellipses. Covariate-adjusted PERMANOVA (adjusting for age, study location, and cohort) indicates a significant group effect (p = 0.001). (**C**) Stacked bar chart of phylum-level taxonomic composition for Control and Insomnia groups. Taxa with relative abundance ≥ 0.1 % and prevalence ≥ 10 % are retained, remaining taxa are grouped as “Other”. (**D**) The Bacillota:Bacteroidota ratios computed from relative abundance profiles are displayed as boxplots. Ratios were significantly higher in controls (unpaired two-sided *t*-test, p = 0.03). Asterisks denote significance: *p< 0.05, ***p<0.001.

To identify cross-cohort microbial markers with greater statistical power and reproducibility, we performed a meta-analysis of five publicly available human fecal 16S rRNA case-control datasets (Fig. 1). All raw data were reprocessed using a single standardized workflow to yield a robust cohort of 468 participants (304 controls and 164 with insomnia). Their respective cohorts and detailed metadata are summarized in Table 1 and **Supplementary Table 1**. The curated list of taxa previously reported as insomnia-associated in the reference papers is provided in **Supplementary Table 2**. Using this combined dataset, we conducted a re-analysis to first assess microbial alpha diversity with the Shannon index to determine whether observed group differences were consistent across studies rather than driven by any single cohort. Our meta-analysis revealed that the insomnia group had a significantly higher Shannon index compared to the control group (p=0.003), indicating that gut microbial diversity is increased in individuals with insomnia (Fig. 2**A**). This trend was consistently observed across all original cohorts. Next, we analyzed community similarity using Principal Coordinates Analysis (PCoA). The results revealed a partial separation between the insomnia and control groups (Fig. 2**B**). This limited separation is likely attributable to heterogeneity introduced by combining multiple experiments and variability across the original studies. Thus, we applied Permutational Multivariate Analysis of Variance (PERMANOVA) to test whether the observed community differences reflected a genuine group effect rather than being driven by potential confounding variables. After controlling for potential confounding factors such as age, study location, and cohort, the group effect remained highly significant (p=0.001), supporting the robustness and reproducibility of these findings (Fig. 2**B**). We next examined community composition (Fig. 2**C**) and found phylum-level differences mainly in Bacillota and Bacteroidota, with significant shifts in their relative abundances between insomnia and control groups (p=0.03; Fig. 2**D**). The ratio is widely used as an ecological indicator of overall community configuration. The observed shift in our analysis likely reflects broad ecological restructuring rather than a disease-specific signature, serving as a coarse descriptor of community organization (Fig. 2**D**). These findings confirm that the gut microbiome of insomnia patients differs structurally from that of healthy controls. The community-level differences observed across multiple studies highlight the need for taxa-focused analyses to identify specific microbial drivers of insomnia. Accordingly, the following sections describe a taxa-focused framework designed to identify these drivers while minimizing cohort-specific effects.

**Table 1:** Characteristics of included insomnia case-control cohorts.

Study title	Sample size (Control / Insomnia)	Degographic detail (Mean age)	Geographic location (Study location)	Insomnia diagnostic criteria*	Reference
Gut microbiota as a diagnostic biomarker for insomnia disorder	10 / 10	29.5	China	DSM-5	(10)
Gut microbiota variation and sleep quality in older adults with insomnia	152 / 72	73.2	Israel	PSQI	(12)
Gut–oral microbiota and serum metabolites in insomnia disorder	59 / 76	33	China	DSM-5	(27)
Gut microbiota and functional brain connectivity in chronic insomnia	34 / 30	39.5	China	DSM-5	(28)
Gut microbiota profiling in paradoxical vs. objective insomnia	78 / 18	68.7	Italy	ICSD-3	(29)

⁎ DSM-5, Diagnostic and Statistical Manual of Mental Disorders, Fifth Edition; ICSD-3, International Classification of Sleep Disorders, Third Edition; PSQI, Pittsburgh Sleep Quality Index (questionnaire-based sleep quality assessment, commonly using a cutoff > 5).

### Conserved microbial taxa signatures: eight taxa are associated with insomnia across multiple statistical analyses

**Fig. 3:**
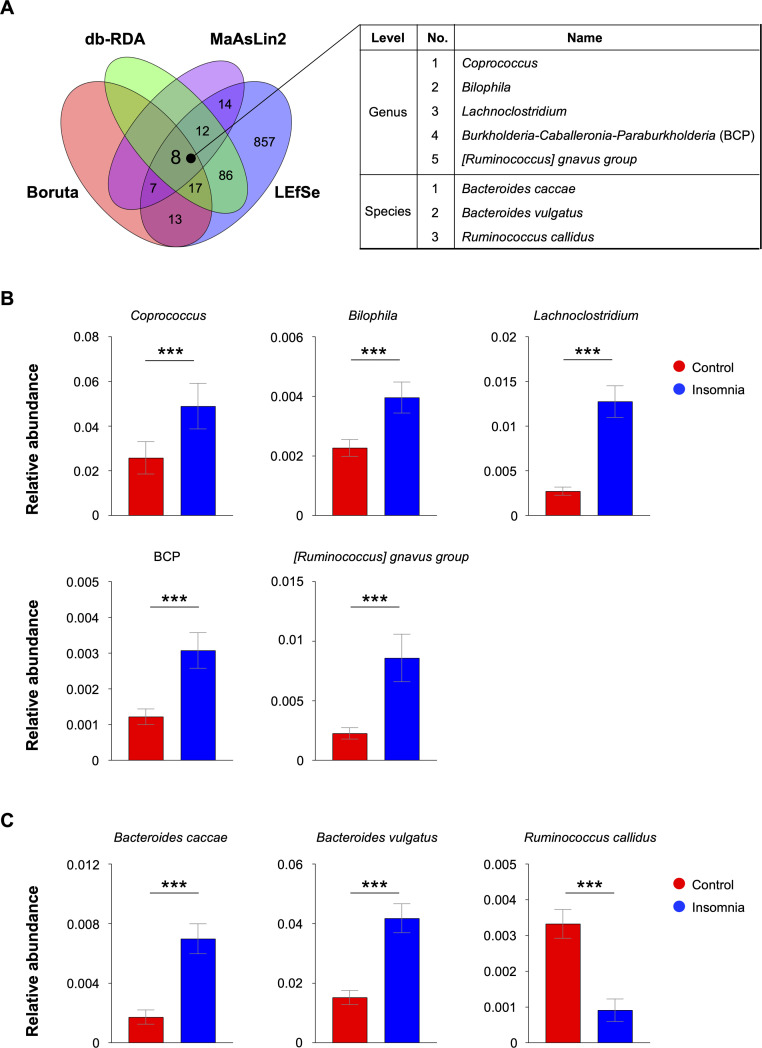
**Eight cross-cohort taxa consistently distinguish insomnia from controls.** (**A**) Overlap across four methods, Boruta, distance-based redundancy analysis (db-RDA), Multivariable Association with Linear Model2 (MaAsLin2), and Linear discriminant analysis Effect Size (LEfSe), after adjusting for age, study location, and cohort. Eight taxa were identified by all four methods. Genus- (B) and species-level (C) relative abundances for the eight consensus taxa are shown as boxplots with individual data points. Boxes represent the median and interquartile range. Group differences were evaluated using two-sided Wilcoxon rank-sum tests. Asterisks denote significance: ***p< 0.001.

To investigate the relationship between microbial communities and insomnia, we employed four distinct approaches to identify taxa associated with this sleep disorder: Boruta feature selection (45 taxa), distance-based redundancy analysis (db-RDA 123 taxa), MaAsLin2 multivariable regression (41 taxa), and LEfSe effect-size screening (1014 taxa). The complete list of candidate genera and their support across the four analyses is provided (**Supplementary Table 3**). Our results revealed a core set of eight conserved taxa significantly associated with insomnia across all four approaches (Fig. 3**A**). Cross-validation among four methods identified eight robust taxa conserved across analyses. Further examination of these shared taxa revealed that five genera were represented in individuals with insomnia: *Coprococcus, Bilophila, Lachnoclostridium*, the *Burkholderia-Caballeronia-Paraburkholderia* complex (BCP), and the *Ruminococcus (*R.*)* gnavus group (Fig. 3**B**). All five genera identified at the genus level were significantly more abundant in individuals with insomnia compared to controls (q≤ 0.05), suggesting potential roles for these taxa in the pathophysiology of insomnia. At the species level, *Bacteroides (*B.*) caccae* and *B. vulgatus* were significantly enriched in insomnia, while *R. callidus* was more abundant in controls; all three associations remained significant after controlling for false discovery in pooled analyses (Fig. 3**C**). These findings collectively support the hypothesis that specific microbial taxa may contribute to the microbiome signatures associated with insomnia. The conserved signature of taxa identified here provides a taxonomic basis for evaluating whether insomnia-related genera are temporally and spatially correlated with consistent predicted functional change.

### Integrated functional-taxonomic analysis reveals coordinated pathway shifts driven by a narrow subset of insomnia-associated taxa

**Fig. 4:**
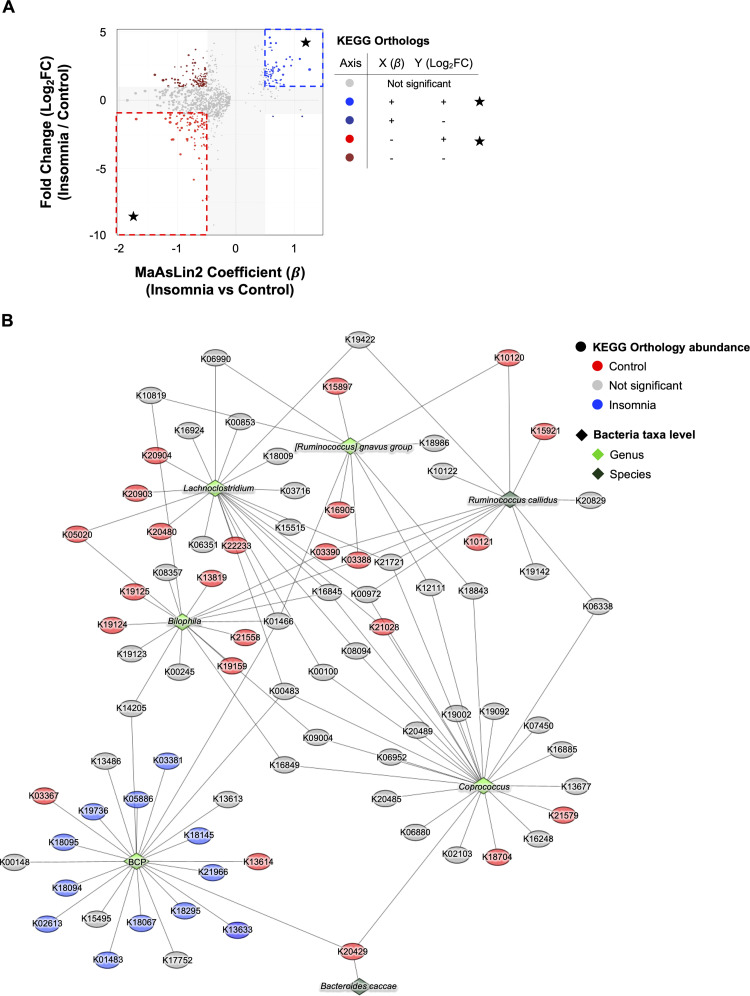
**Predicted functional signatures align with insomnia-associated taxa.** (A) The scatterplot compares the MaAsLin2 regression coefficient (β, between Insomnia and Control) on the horizontal axis and the unadjusted log2 fold change (log_2_FC; Insomnia vs Control) on the vertical axis for KEGG orthologs (KOs) predicted by PICRUSt2. The regression coefficient (β) represents the covariate-adjusted association between KO abundance and insomnia status. Analyses were performed on CLR-transformed KO abundance tables, and the MaAsLin2 models included age, study location, and cohort as covariates. Each point represents a single KO. The thresholds were applied (|β| ≥ 0.5, |log_2_FC| ≥ 1, and FDR-adjusted q≤ 0.05). KOs exceeding both |β| and |log_2_FC| thresholds are shown in the white region, whereas those not exceeding these thresholds fall within the gray region. Colors indicate the direction and agreement between the regression coefficient (β) and the log_2_FC. Red dots indicate KOs with a negative regression coefficient (β< 0, lower abundance in insomnia; highlighted with the red box), whereas blue dots indicate KOs with a positive regression coefficient (β>0, higher abundance in insomnia; highlighted with the blue box). Burgundy and navy dots indicate discordant directionality between the regression coefficient (β) and the log_2_FC. Gray dots mean non-significant features. (**B**) The KO-taxa association network depicts how the eight consensus taxa contribute to the KEGG orthologs that were significantly associated with insomnia or control groups based on both the MaAsLin2 coefficient and the log2 fold change. Contributions were estimated using PICRUSt2, and an edge is drawn between a taxon and a KO when the taxon accounts for at least 0.1 % of the predicted abundance of that KO. Edge colors follow the direction of the KO, so that control-enriched KOs appear in red and insomnia-enriched KOs appear in blue, as determined by the group differences identified through the two-sided *t*-test. KO nodes are drawn as ovals to distinguish them from taxa, and their colors reflect the significant directionality of the KO between the two groups. Taxa are drawn as diamonds, and their colors represent genus-level differential abundance direction. Node labels show KO identifiers or genus names. A force-directed layout organizes the network so that KOs form functional clusters and taxa are positioned according to their contribution patterns, illustrating how the eight taxa collectively support the functional signatures associated with insomnia.

To determine whether shifts in microbial metabolic potential accompanied the taxonomic alterations observed in insomnia, we predicted KEGG Ortholog (KO) abundances using PICRUSt2 and modeled group-level effects (insomnia and control groups) by using MaAsLin2 while adjusting for age, study location, and cohort. To validate the robustness of these associations, we compared the adjusted Group coefficients from MaAsLin2 with the corresponding simple log2 fold changes (log_2_FC) calculated from the same samples, and retained KOs that were significant after multiple testing correction and directionally consistent with the log_2_FC (**Supplementary Table 4**). This integrative filtering identified 151 functionally robust KOs: 31 KOs were significantly enriched in the insomnia group, whereas 120 KOs were significantly depleted (Fig. 4**A**). These results demonstrate that the impact of insomnia on the gut microbiome extends beyond compositional changes and involves coordinated shifts in predicted metabolic capacity.

To identify microbial taxa responsible for the observed functional changes (KO pathways), we integrated PICRUSt2 contribution scores with the eight covariate-adjusted taxa that were identified earlier (Fig. 3**A**) and constructed a bipartite taxon-KO network (Fig. 4**B**). Seven of the eight taxa exhibited nonzero functional contributions, whereas *B. vulgatus* showed no detectable input to any KO identified in this study. Across the contributing taxa, we identified 81 predicted KO functions, including 21 with significant group-level differences (Fig. 4**B**). Taxa *Coprococcus, Bilophila, Lachnoclostridium*, the BCP complex, the *R. gnavus* group, *B. caccae*, and *R. callidus* were primarily associated with control-enriched or non-significant KOs, indicating only marginal involvement in insomnia-associated functional shifts (**Supplementary Table 5**). In contrast, the BCP complex emerged as the central insomnia-associated hub: it was the only taxon contributing to all 12 disease-enriched KOs and provided for the majority of functional input within the insomnia-enriched cluster. Collectively, these results indicate that insomnia-linked functional alterations are concentrated rather than community-wide, converging on a narrow subset of taxa, with the BCP serving as the dominant contributor to insomnia-associated functional shifts.

### Correlation analyses independently validate taxon-KO interactions and highlight the BCP complex as the major contributor to insomnia-associated functions

**Fig. 5:**
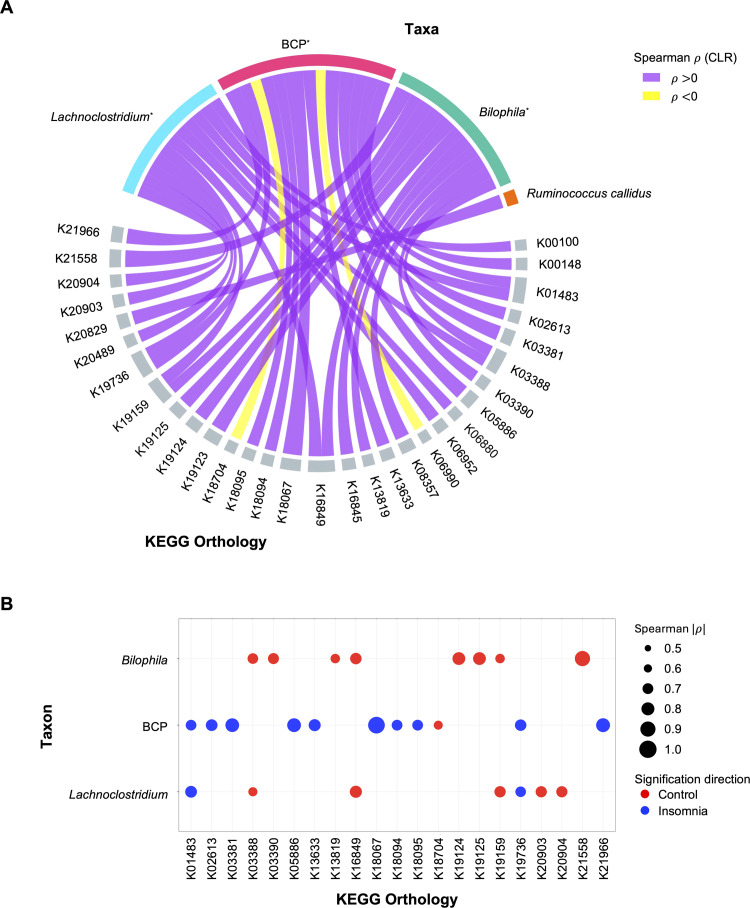
**Independent correlation analyses validate the taxon-KO linkages identified in the functional network**. (**A**) The clustered chord diagram displays significant Spearman correlations between taxa and KEGG orthologs. Correlations were assessed using Spearman’s rank correlation. Benjamini–Hochberg false discovery rate (FDR) procedure was used for multiple testing. Associations were considered significant at q≤ 0.05 with an absolute correlation coefficient |ρ| ≥ 0.5. Links are colored according to the sign of the correlation coefficient, with positive correlations shown in purple and negative correlations shown in yellow. Taxa are assigned distinct colors, and all KEGG orthologs are shown in gray. Asterisks next to taxon names indicate cases in which the direction of the taxon KO association matches the direction observed in the functional network (Fig. 4**B**). (**B**) The dot plot summarizes the significant taxon-KO associations. Each point represents a taxon-KO pair, and the size of the point corresponds to the absolute correlation coefficient. Colors indicate KO directionality based on the MaAsLin2 coefficient and the log2 fold change, where red represents lower abundance in insomnia and blue represents higher abundance in insomnia.

We validated the taxon-KO relationships identified in the bipartite network by performing pairwise Spearman correlation analyses between KO abundances and the eight covariate-adjusted taxa (Fig. 5**A** and **Supplementary Table 6**). This analysis confirmed that a subset of network-derived associations was supported by direct covariation in the abundance data. Four taxa, *Bilophila*, the BCP complex, *Lachnoclostridium*, and *R. callidus*, showed reproducible associations with subsets of the network-derived KOs (q≤0.05). In particular, *Lachnoclostridium* exhibited significant positive associations with K20903 and K20904, which encode the α and β subunits of (R)-2-hydroxyglutaryl-CoA dehydratase (EC:4.2.1.167), involved in anaerobic glutamate degradation (**Supplementary Table 6**). *Bilophila* displayed significant correlations with K03388 and K03390, annotated as heterodisulfide reductase (HDR) complex subunits that are involved in anaerobic redox metabolism and support fermentative processes through redox balancing, including pathways associated with short-chain fatty acid (SCFA) metabolism (**Supplementary Table 6**). *Bilophila* was also associated with K21558, encoding a CRP/FNR-family transcriptional regulator, and with K19124, K19125, and K19159, which correspond to CRISPR-associated Cascade subunits and a toxin–antitoxin component, respectively. *R. callidu*s showed moderate correlations across several KOs, without a dominant functional cluster. Interestingly, the BCP complex demonstrated the broadest and most diverse correlation profile, including K01483 (ureidoglycolate lyase, EC:4.3.2.3), K02613 (phenylacetyl-CoA epoxidase subunit PaaE), K05886 (NADP-dependent serine dehydrogenase, EC:1.1.1.276), K18067 (phthalate 4,5-dihydrodiol dehydrogenase, EC:1.3.1.64), collectively implicating nitrogen/amino-acid metabolism and aromatic compound processing (**Supplementary Table 6**). Notably, PaaE (K02613) is a core component of the phenylacetate degradation pathway, whose metabolites have been previously implicated in host signaling contexts (**Supplementary Table 6**). Additional correlations involved K18094 and K18095, which encode components of multidrug efflux systems, K19736, a TetR/AcrR-family transcriptional regulator, and K21966, Mat/Ecp fimbrial outer-membrane usher protein (**Supplementary Table 6**). These associations span metabolic, regulatory, and cellular stress-adaptation functions.

We further visualized all significant taxon–KO correlations for *Bilophila*, the BCP complex, and *Lachnoclostridium* using a dot plot, integrating effect size, directionality, and reproducibility across analyses (Fig. 5**B**). This representation revealed a pronounced concentration of high-magnitude, directionally consistent effects within a limited subset of taxa, resolving heterogeneity in functional associations that was not fully apparent from the bipartite network or correlation analyses alone. Notably, the BCP complex accounted for the largest proportion of significant combined KOs identified through integrated analyses of KO–bacteria contributions and correlations (52.4 %, 11/21 KOs), with consistently aligned effect directions (90.9 %, 10/11 KOs) enriched in the insomnia group, whereas other taxa showed more sparse or variable association patterns (Fig. 5**B**). Together, these patterns indicate that insomnia-associated functional variation is driven by a concentrated subset of taxa rather than broadly distributed across the community.

## Discussion

In this study, we performed a cross-cohort meta-analysis of publicly available human fecal 16S rRNA datasets to test whether insomnia is associated with reproducible gut microbiome alterations. By harmonizing raw sequencing data across studies and controlling for cohort-specific effects, we reveal robust, cross-cohort microbial signatures of insomnia that persist despite substantial technical and population heterogeneity. Despite modest global differences in community composition, insomnia was marked by directionally consistent and statistically robust microbial alterations across cohorts, supporting a model of conserved, pathway-specific microbiome remodeling rather than nonspecific dysbiosis. Notably, these alterations extended beyond taxonomic composition to predicted functional profiles, which converged on a limited subset of taxa. Together, these findings provide a cohesive framework for exploring how specific microbial features may contribute to physiological processes linked to insomnia.

Importantly, the observed functional signatures indicate targeted metabolic remodeling rather than broad microbiome dysbiosis (Lloyd-Price et al., 2017). This remodeling is centered on a small number of anaerobe-associated taxa, whose predicted functions implicate shifts in redox balance, fermentative metabolism, and short-chain fatty acid–related pathways (Fischbach and Sonnenburg, 2011; Louis and Flint, 2017; Manor and Borenstein, 2017). Correlation-based validation further demonstrated that insomnia-associated functional variation is concentrated within a limited subset of taxa, most prominently the BCP complex, rather than being diffusely distributed across the microbial community (Franzosa et al., 2014). This convergence across independent analytical approaches supports a model in which focused microbial functional niches, rather than global compositional shifts, underlie insomnia-associated microbiome signals (Louca et al., 2018). Among the taxa identified, *Lachnoclostridium* showed reproducible associations with K20903 and K20904, encoding enzymes involved in anaerobic glutamate fermentation (Buckel and Thauer, 2013; Kanehisa et al., 2021). Although this pathway does not represent a canonical route for butyrate synthesis, glutamate-derived fermentative metabolism can intersect with short-chain fatty acid (SCFA)–associated networks by sustaining redox balance and carbon flow within anaerobic communities (Vital, Howe and Tiedje, 2014; Neis, Dejong and Rensen, 2015). *Bilophila* exhibited associations with heterodisulfide reductase (HDR) complex subunits, which play central roles in anaerobic redox metabolism and electron-balancing processes that support fermentative flux, thereby linking redox-coupled metabolism to fermentative pathways associated with short-chain fatty acid (SCFA) metabolism (Thauer et al., 2008; Buckel and Thauer, 2013). These associations are consistent with redox-coupled metabolic activity under anaerobic conditions rather than representing direct evidence of redox stress adaptation. Within the BCP complex, correlated functions spanned nitrogen and amino-acid metabolism, aromatic compound degradation, and cellular stress adaptation (Fuchs, Boll and Heider, 2011; Parks et al., 2018; Tofani et al., 2025). Notably, phenylacetyl-CoA epoxidase (PaaE) and related enzymes participate in aromatic substrate processing, yielding metabolites that, while not classical signaling molecules, have been implicated in indirect modulation of host physiological contexts (Dodd et al., 2017; Teufel et al., 2010). Taken together, these results support a model in which insomnia-associated microbiome signals arise from constrained metabolic niches with potential relevance to host neurophysiology, rather than from generalized community-level perturbation.

Beyond metabolism, the BCP complex was enriched for functions associated with cellular stress adaptation and regulatory control, including multidrug efflux systems (K18094, K18095), TetR/AcrR-family regulators (K19736), CRP/FNR-family transcriptional regulators (K21558), and the YefM antitoxin (K19159) (Green and Paget, 2004; Gerdes, Christensen and Lobner-Olesen, 2005; Cuthbertson and Nodwell, 2013; Li, Plesiat and Nikaido, 2015). These systems are widely implicated in bacterial persistence under inflammatory, oxidative, and bile-acid–rich environments and are characteristic of microbes adapted to dynamic host interfaces (Begley, Gahan and Hill, 2005; Donaldson, Lee and Mazmanian, 2016; Fang et al., 2016). Importantly, metabolic redox balancing under anaerobic conditions can also support microbial persistence and functional stability in immune-active mucosal environments without implying a classical stress-response phenotype (Thauer et al., 2008). Their coordinated presence suggests an enhanced capacity for responding to immune pressure and fluctuating redox conditions within the gut mucosa (Winter et al., 2013; Lopez et al., 2015). Low-grade immune activation and altered barrier signaling are well-documented features of chronic insomnia, linking sleep disruption to systemic inflammation (Mullington et al., 2010; Irwin and Opp, 2017; Besedovsky, Lange and Haack, 2019). Consistent with this host-associated persistence, microbial functions that enable stress tolerance, redox-responsive regulation, and controlled growth arrest may indirectly interact with host immune tone, reinforcing bidirectional communication at the intestinal interface (Round and Mazmanian, 2009), reflecting ecological adaptation to fluctuating mucosal conditions rather than a dedicated redox stress–response phenotype.

Our study identifies reproducible and functionally coherent microbiome signatures associated with insomnia, yet several limitations should be considered. A key limitation is that PICRUSt2-derived functions represent predicted metabolic potential from 16S data and do not directly reflect measured gene expression or metabolic activity in vivo. Accordingly, the functional shifts described here should be interpreted as putative alterations in metabolic potential that warrant validation using shotgun metagenomic, metatranscriptomic, or metabolomic approaches. In addition, although active psychotropic medications were excluded at the time of sampling in the original cohorts, information on other lifetime medication exposures was not consistently available, and unmeasured medication effects may have contributed to the observed associations. Beyond these methodological and clinical considerations, the ecological interpretation of the BCP complex warrants careful consideration. Members of this complex are primarily environmental bacteria inhabiting soil, water, and plant-associated niches and are not typically dominant commensals of the healthy human gut (Parks et al., 2018). Certain Burkholderia species function as opportunistic pathogens, most commonly in immunocompromised hosts or in cystic fibrosis lung (Li, Plesiat and Nikaido, 2015; Fang et al., 2016), and clinical manifestations are more frequently reported in the respiratory tract than in the gastrointestinal system (Mahenthiralingam, Urban and Goldberg, 2005; Folescu et al., 2015). BCP genomes exhibit broad metabolic versatility, including aromatic compound degradation, xenobiotic processing, and stress-responsive regulatory systems (Parks et al., 2018). In this study, the presence of BCP at differentially detectable levels in the gastrointestinal system may reflect context-dependent expansion or detectability within the gut, with potential implications for localized metabolic or immune modulation under particular environmental or lifestyle conditions. Community-level findings should also be interpreted within a broader ecological context. Reduced alpha diversity is widely reported across many inflammatory and metabolic diseases and is often considered a hallmark of dysbiosis (Lozupone et al., 2012; Lloyd-Price et al., 2017). In our analysis, Shannon diversity was increased in insomnia. This trend was consistent with all five original studies, with two reaching statistical significance. When integrating all studies under a unified analytical framework, the modest increase in Shannon diversity became statistically significant. This suggests that insomnia-associated microbial alterations may reflect subtle ecological redistribution within the community rather than the canonical diversity-loss model of inflammatory disease. Finally, the proposed mechanistic links remain indirect and inferential. Many studies indicate that microbiome–host interactions in sleep and neuroimmune regulation are largely indirect and mediated through metabolic and immune pathways. Altered microbial processing of aromatic amino acids, including phenylacetate degradation, has been linked to the production of immunomodulatory and neuromodulatory metabolites (Dodd et al., 2017). Given that chronic insomnia is associated with low-grade systemic inflammation (Irwin and Opp, 2017; Besedovsky, Lange and Haack, 2019), these observations support a mechanistic link between microbial metabolism, neuroimmune inflammatory processes, and chronic insomnia. Accordingly, the predicted metabolic functions identified in our study, including redox-balancing and short-chain fatty acid–related pathways, may intersect with immunomodulatory and neuromodulatory processes that represent established features of insomnia pathophysiology. Given their basis in predicted functional profiles and associative analyses, they should be considered hypothesis-generating, and their clinical specificity remains uncertain without direct host physiological validation.

Taken together, our findings support an indirect model linking gut microbial activity to insomnia-associated host states through metabolic and immune contexts. The BCP complex, in particular, appears to integrate redox-coupled fermentation, aromatic substrate processing, and stress-adaptive regulation, positioning it as a key functional mediator within an ecosystem shaped by sustained arousal and immune perturbation. Future studies incorporating targeted metabolomics, immune profiling, and longitudinal sleep phenotyping will be essential to test these indirect pathways and establish causal relationships.

## Funding

This research was supported by a grant of the Korea Health Technology R&D Project through the Korea Health Industry Development Institute (KHIDI), funded by the Ministry of Health & Welfare (HI19C1085) and Global-Learning & Academic Research Institution for Master’s-PhD students, and Postdocs (G-LAMP) Program of the National Research Foundation of Korea (NRF) grant funded by the Ministry of Education (No. RS-2023-00301938).

## Data and code availability

All raw sequencing data are available from the original public repositories cited in **Supplementary Table 1**.

All scripts used for preprocessing, statistical modeling, and figure generation are available at: https://github.com/Sun980610/26_Insomnia_meta-analysis

## Conflict of interest

The authors declare that they have no conflict of interest.

## Declaration of competing interest

The authors declare that they have no known competing financial interests or personal relationships that could have appeared to influence the work reported in this paper.

## Appendix Supplementary materials

Image, application 1
